# Internet-of-Things Devices in Support of the Development of Echoic Skills among Children with Autism Spectrum Disorder

**DOI:** 10.3390/s21134621

**Published:** 2021-07-05

**Authors:** Krzysztof J. Rechowicz, John B. Shull, Michelle M. Hascall, Saikou Y. Diallo, Kevin J. O’Brien

**Affiliations:** 1 Virginia Modeling, Analysis & Simulation Center, Old Dominion University, Suffolk, VA 23435, USA; jshull@odu.edu (J.B.S.); sdiallo@odu.edu (S.Y.D.); kobrien@odu.edu (K.J.O.); 2 Mea’Alofa Autism Support Center, Chesapeake, VA 23321, USA; mhascall@learnwithmasc.org

**Keywords:** autism, internet-of-things, language therapy, human-computer interactions

## Abstract

A significant therapeutic challenge for people with disabilities is the development of verbal and echoic skills. Digital voice assistants (DVAs), such as Amazon’s Alexa, provide networked intelligence to billions of Internet-of-Things devices and have the potential to offer opportunities to people, such as those diagnosed with autism spectrum disorder (ASD), to advance these necessary skills. Voice interfaces can enable children with ASD to practice such skills at home; however, it remains unclear whether DVAs can be as proficient as therapists in recognizing utterances by a developing speaker. We developed an Alexa-based skill called ASPECT to measure how well the DVA identified verbalization by autistic children. The participants, nine children diagnosed with ASD, each participated in 30 sessions focused on increasing vocalizations and echoic responses. Children interacted with ASPECT prompted by instructions from an Echo device. ASPECT was trained to recognize utterances and evaluate them as a therapist would—simultaneously, a therapist scored the child’s responses. The study identified no significant difference between how ASPECT and the therapists scored participants; this conclusion held even when subsetting participants by a pre-treatment echoic skill assessment score. This indicates considerable potential for providing a continuum of therapeutic opportunities and reinforcement outside of clinical settings.

## 1. Introduction

Internet-of-Things (IoT) devices are becoming pervasive in our society. There are over 30 billion IoT devices, many of which are in our houses and cars [1]. IoT technology has the potential to assist people with disabilities, which includes individuals with autism spectrum disorder (ASD), in living independent and productive lives.

Currently, most people diagnosed with ASD see improvement in condition-related symptoms as they transition into adolescence or early adulthood [2]. However, long-term outcomes, despite existing assistance and support programs, can be considered unsatisfactory [3,4,5]. The underwhelming statistics also include over half of those with higher cognitive abilities (non-verbal IQs > 70) [6]. However, despite occasional discouraging results, proven techniques (self-monitoring, video-modeling, and individual work systems) for mitigating the effects of the removal of adult support (supervision, prompting, and contingencies) exist [7]. These techniques shift stimulus control from the supporting adult to the individual, thus, promoting agency and increasing independence.

Although these techniques do not produce the same quality of outcomes in all individuals with ASD, current limitations preventing extending such solutions to most subjects could be overcome with creativity and ingenuity [7]. Such a solution or a component thereof can be seen in digital voice assistants (DVAs), such as Amazon Alexa. Anecdotally, many special needs parents already use DVAs to help their children with autism spectrum disorder improve their verbal skills [8]. Caregivers and stakeholders who support independent living preparedness envision IoT devices as one of the main elements of systems in providing a continuum of therapeutic opportunities and supporting independent living [9].

This study aimed to examine the accuracy of a DVA in recognizing utterances from children with ASD during a therapeutic session. To achieve this objective, we built an Alexa Skill and deployed it at a local center during therapy sessions aimed at improving the verbal and echoic skills of a group of children with ASD. Based on 979 prompts made by the DVA and the corresponding participants’ responses or the lack thereof, we were able to determine no significant difference between the scores made by the AI-enabled speaker and the therapist.

The outline of the paper is as follows: Section 1 an introduction to the paper; Section 2, related work; Section 3, materials and methods; Section 4, results; Section 5, the discussion; and our conclusions in Section 6.

## 2. Related Work

In recent years, the IoT and its applications to many domains of our lives, such as healthcare, manufacturing, and transportation, have received a great deal of researchers’ attention [10,11]. Although people with disabilities can also benefit from some of the advances stemming from that research, few studies have investigated whether IoT devices, and more specifically DVAs, have the potential to be increasingly beneficial for people with language-development challenges.

A number of scholars have investigated how using computers can complement the speech therapy of children with ASD. For the most part, each of them concluded that there is no one-size-fits-all design of educational tools due to the range of differences between children [12,13,14,15,16]. Recent work by Porayska-Pomsta et al. [17] compared interactions between children who had ASD with an artificial intelligence (AI) algorithm and a therapist. These findings indicated that children were more responsive to human practitioners; however, as the study progressed, there was an overall increase in responsiveness to both the AI and the teacher.

In [18], the authors reminded us of the qualities of computer-based tutors—patience and adaptive responses. They conducted a study with children with ASD (aged 3–6 years) to investigate whether the children were more attentive, recalled more nouns, and were more motivated when they interacted with the computer rather than with the therapist. Their result suggests that DVAs could deliver similar benefits, regardless of the small sample size.

Vyshedskiy et al. [19] focused on mitigating the impact of prefrontal synthesis (PFS) paralysis on language in children with ASD through verbal and non-verbal exercises delivered through a mobile application called MITA. The exercises, aimed at improving the ability to consciously synthesize novel mental pictures, significantly enhanced the receptive and expressive language scores in children with ASD aged 2–12 years. Among potential reasons for the more significant improvement in the receptive language score, the authors proposed the prosody stability of computerized language therapy, a substantial cost associated with the therapists’ frequent availability, and high accessibility of the application to parents who can administer MITA at when the child is receptive and in a good mood. Similar benefits could be observed while utilizing a DVA to supplement therapy.

Allen et al. [20] investigated the accuracy of phrase recognition of an Alexa-based DVA spoken by a speech-language pathologist (SLP) in the context of therapy aimed at children with ASD. The reported accuracy was low (almost 9%) and increased to over 45% when the relevant phrases were uploaded to augment the skill’s vocabulary. Allen et al. acknowledged that the main drawback was the requirement of speaking the exact phrase to solicit the DVA’s response. This suggests that a skill used for therapeutic purposes should leverage a predefined list of utterances and allow flexibility (or eliminate the requirements) in how the phrase is structured.

Yu et al. [21] investigated whether SLPs with experience working with ASD populations could consistently speak a phrase that triggered a DVA, the Echo Show^TM^. DVAs were evaluated on the number of correct visual material associated with the phrases, and the SLPs completed a questionnaire in which their opinions on the DVA as a potential clinical tool were captured. The data provided preliminary evidence that the number of syllables or phrase lengths had no impact on the DVA’s response accuracy. In certain rare cases, specific words or phrases did impact the accuracy; in these cases, there were workaround strategies developed. The SLPs indicated that they would be willing to use the DVA in their practice. An interesting finding was that a majority of the SLPs pointed to the DVA as a potential therapeutic tool for people with ASD to increase their independence when used at home.

Pradhan et al. [22] investigated how accessible the line of Alexa-based DVAs (Echo, Echo Dot, and Tap) were by analyzing the content of reviews written on Amazon’s website by people with cognitive, sensory, and physical disabilities. Over 13% of the reviews mentioned speech impairment, almost 12% cognitive impairment, and almost 5% use by a child. Several reviews written by people with speech impairment reported better accuracy of speech recognition compared with other voice programs and, in some cases, compared with other humans. Some reviewers, cited in [22], found the interactions with the DVA therapeutic by helping them or their caretakers to talk more proficiently. One reported review dealt with an autistic child using the DVA to practice speech while his parents perceived improvement when the device understood the command.

Language attitudes toward DVAs are essential in deciding how successful interactions between humans, including those with ASD, and AI, are. Cohn et al. experimented to determine whether people attributed similar language attitudes to Amazon’s Alexa and a human speaker. The authors also administered the Autism Quotient (AQ) survey [23] to investigate whether there were patterns of individual variations in these ratings. In general, the study participants rated Alexa’s voice as intelligent, professional, and attractive as the human’s voice, and, contrary to the researchers’ expectations, they found Alexa more likable. In contradiction to some studies [24], the authors did not observe a relationship between higher AQ scores and a variation in ratings. However, the subjects in the study were undergraduate students without a formal ASD diagnosis, which may explain the results.

## 3. Materials and Methods

The institutional review board of the Old Dominion University approved the study protocol. The participants were identified by a recruiter who was not part of the research team. Since all participants were minors, written informed consent was obtained from their parents and legal guardians. The study followed the Strengthening the Reporting of Observational Studies in Epidemiology (STROBE) reporting guidelines [25].

### 3.1. Autism Spectrum Engaged Cloud Technology

We worked with an experienced Board-Certified Behavior Analyst to develop a custom-made Amazon Alexa skill called Autism Spectrum Engaged Cloud Technology (ASPECT). In the skill design stage, we applied 18 out of 20 guidelines for researchers and developers providing software solutions for children with ASD [26]. To illustrate, we started the process by attending a local therapy center and viewing the session recordings focused on practicing echoic skills (Guideline #1).

The application of Guidelines #3–6 resulted from the requirements of the IRB process, including parental consent and the protocol followed at the end of the trials. While designing ASPECT, we did not consider adaptation and customization (Guideline #16) in this skill version. ASPECT does not accommodate special interests in the skill functionality (Guideline #17). However, it is up to the therapist to select utterances that correspond to the child’s echoic proficiency level. See the Supplementary Materials for detailed information about the guidelines applied to ASPECT.

ASPECT replicates how a therapist helps students practice their verbal and echoic skills, including prompts and feedback. ASPECT was developed within the Amazon Web Services framework using a set of Lambda functions and Amazon’s automatic speech recognition (ASR) engine [27,28,29,30]. Using the framework, similarly to [20], we built a custom intent model based on a set of 357 utterances (please see the supplement for the list of utterances) that were used to practice verbal and echoic skills. An utterance is a word or a small group of words standing together as a conceptual unit. For each session, the therapist can either select utterances from this list or add new utterances of their choosing.

**Figure 1:**
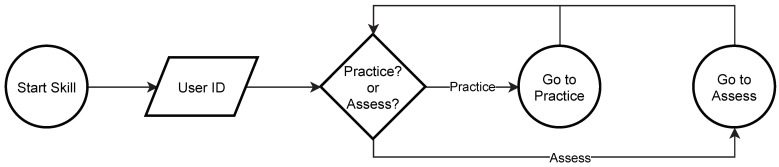
ASPECT start-up mode flowchart.

When the ASPECT skill is selected by the therapist, first, it enters into the startup mode. The therapist is asked to provide the unique identifier associated with the particular participant to anonymize the data. Next, either a practice or assessment mode can be selected. The startup process is outlined in Figure 1.

**Figure 2:**
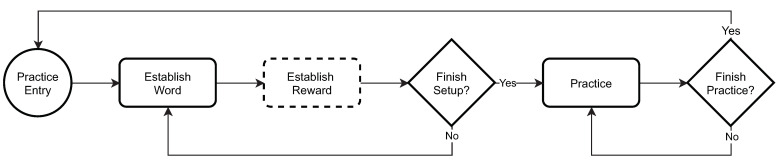
ASPECT practice mode flowchart.

The practice mode, outlined in Figure 2, is designed to familiarize a child with (1) the Alexa Echo device, (2) the ASPECT skill, and (3) the overall setup of a session. In this mode, ASPECT allows therapists to select one-syllable utterances that a child can practice outside their therapy session. This includes utterances that the child is familiar with or utterances that they need to work on to improve their verbal and echoic skills. Although ASPECT allows custom rewards (e.g., a favorite song), we did not leverage this functionality in the study and every child received positive feedback or encouragement to try again.

**Figure 3:**
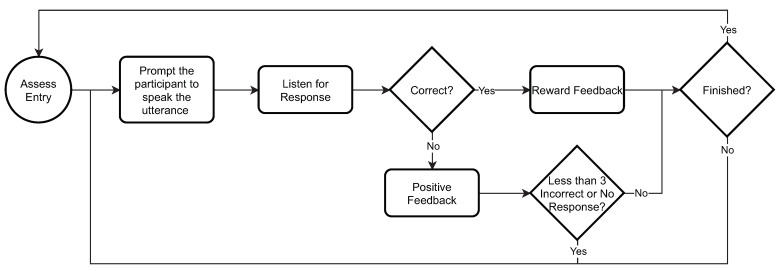
ASPECT assessment mode flowchart.

The assessment mode, outlined in Figure 3, similarly to the practice mode, starts with the setup, which allows the therapist to prepare a session by specifying a set of utterances that they would like the child to practice during a therapy session. Once the utterances are set and the session begins, ASPECT prompts the child by saying an utterance and waits for the child to repeat it. ASPECT provides positive feedback if it scores the child’s response as “correct” or asks them to try again up to three times if scores the answer as “incorrect” or “no response”.

ASPECT is hosted in the public cloud and does not store any personal information other than the participant’s randomly assigned ID.

### 3.2. Study Design

A group of children from the patient population of a local autism support center diagnosed with ASD was identified and recruited for participation in the study. Each child’s ability to repeat speech sounds was measured by the Early Echoic Skills Assessment (EESA) test, which is part of the Verbal Behavior Milestones Assessment and Placement Program (VB-MAPP) [31]. VB-MAPP is an assessment tool used with people diagnosed with ASD and other language delays.

EESA consists of five groups of utterances to echo, which are scored 1, 0.5, or 0 depending on the child’s performance. ESSA scores add up to 100. We also recorded the child’s age and gender at the beginning of the treatment.

Each child enrolled in the experiment interacted with ASPECT in 30 sessions as part of their verbal and echoic communication skills training. Participants practiced interacting with ASPECT before the start of the first therapeutic session to avoid negative reactions and tantrums caused by the introduction of the device. Each therapeutic session started with the therapist verbally entering the utterances that the child would practice. ASPECT stated the first utterance and instructed the student to repeat the utterance. The student had up to three tries to say the utterance correctly.

ASPECT recorded each response from the participant and used Amazon’s ASR to identify it. Each response was logged in a database including when the participant did not respond or if the microphone array could not pick up a response. The therapist and ASPECT independently scored each response as “correct,” “incorrect,” or “no response.” The therapist did not have access to the scores provided by ASPECT at any time during or after the study. Each session was audio recorded by the therapist, and an EESA score was calculated for each child after 30 sessions.

#### Setting

The study was conducted at a local therapy center providing services to families with children diagnosed with ASD. The enrollment started on 15 March 2018 and concluded on 30 September 2019. Data collection started on 30 March 2018 and ended on 17 October 2019.

### 3.3. Participants

Children between ages 1 and 17, diagnosed with ASD, and enrolled at the local therapy center were eligible to participate in the study. To avoid the perception of coercion, a staff member who was not part of the research team identified and approached families whose children met the criteria and explained verbally what the study entailed. She also explained that participation or non-participation would have no effect on their child’s therapy schedule. Families were also verbally informed that they may withdraw from the study at any time without penalty. If the family were interested in participating, they received the consent form from the therapist.

Participants who showed a significant increase in intensity, frequency, or duration of maladaptive behavior [32,33] or exhibited a new maladaptive behavior while working with ASPECT were withdrawn from the study. Participants may also have withdrawn their assent verbally by stating that they did not want to continue.

A total of 23 children were recruited for the study. The recruitment was performed on a rolling basis throughout the study. The participants were assigned to one of three groups. Group 1 had up to 10 children interacting with ASPECT only; Group 2 also had up to 10 children interacting with a therapist only, and Group 3 was a reserve of up to 10 children to fill in as replacements for the first two groups. This study reports only on Group 1. Out of 23 children, we were able to construct a group of nine participants interacting with ASPECT only (Group 1) over 30 sessions. the participants’ age ranged from 4 to 7 years with a mean (SD) age of 5.3 (1.1), with one identified as female and eight as male. Pre-treatment EESA scores ranged from 15.5 to 98.8 with a mean (SD) EESA of 74.2 (29.8).

### 3.4. Bias

ASPECT was built as a custom interaction model (also called a skill) within the AWS framework. The speech recognition engine is designed to map the user’s spoken utterance to an intent. To illustrate, “get a cab,” “find me a ride,” and “I need to get to the airport” would be mapped to an “order a car” intent resulting in dispatching a taxi or rideshare service. This so-called “intent model” is focused more on what the user is trying to achieve, i.e., the intent detection, rather than on how the user pronounces the words. Unlike therapists, ASPECT did not account for prosodic features like stress, intonation, pitch, and rhythm, which could explain some of the discrepancies in scoring answers as “correct” or “incorrect.” A correct response according to ASPECT may be scored as “incorrect” by the therapist if, for instance, the utterance is monotone or lacks emphasis on the right verb.

Therapists can also be biased because they are familiar with the participants’ speaking outside of the sessions and, therefore, are attuned to how they pronounce certain utterances. This familiarity can also bias therapists toward more lenient scoring because (1) they know the participant’s daily workload and are inclined to give them a break because of fatigue, and (2) they are aware from previous sessions that the participant can pronounce the utterance and might be having trouble in that specific session. This bias also might account for some of the mismatches in scoring, especially in cases where the therapist scored a response as “correct” where ASPECT scored it as “no response”.

### 3.5. Data Sources and Measurements

The internal records of the therapy center provided pre- and post-treatment EESA scores, age, gender, and the enrollment date. Therapists provided manual scores of utterances, according to VB-MAPP [31], for all participating children. ASPECT recorded the utterances used in each session, date and time of the session, anonymous participant ID, and whether the ASPECT score of the utterance spoken by the child was “correct,” “incorrect,” or “no response”.

### 3.6. Utterances

During each session, participants were presented with utterances to repeat. The length of the utterances ranged from 1 to 6 words with a mean (SD) length of 3.5 (1.9), and the starting length was correlated with a child’s echoic proficiency. Thirty-six unique utterances were used in all 30 sessions among all participants. The top three utterances used were (114 times): “my new pencil is lost,” “cheese and crackers,” and “look at my new snow boots”.

### 3.7. Statistical Analysis

We employed a frequency analysis to investigate the utterances used in the sessions. Frequency analysis gives us insights into the relative difficulty and the level of proficiency of the participant for each session. As we had categorical data and two types of scorers (ASPECT vs. therapist), we created contingency tables of the ASPECT and therapist scores and used $\chi^{2}$ and Fisher’s exact tests (*p* < 0.05 if dependency exists) to investigate the relationship between the probability of the therapist and ASPECT giving participants the same score. In 294 cases, ASPECT malfunctioned and failed to score due to improper setup or the therapist score was missing because they failed to record it. In those cases, we disregarded the ASPECT–therapist scoring pair. All analyses were conducted using R version 3.6.3 with supplementing packages [34].

## 4. Results

### 4.1. Comparison between the Therapist and ASPECT Scores

In 675 (69%) out of 979 observations, ASPECT scored in the same manner as the therapist (Table 1). ASPECT and the therapist agreed on 203 (66%) out of 307 “correct” scores and 177 (51%) out of 345 “incorrect” scores. On “no response” scores, both agreed in 295 (90%) out of 327 observations. In 72 (7%) observations, ASPECT scored a response as “correct” where the therapist indicated an “incorrect” or “no response” score and, 153 (16%) times, ASPECT failed to register a response where the therapist indicated a “correct” or “incorrect” score.

By performing a $\chi^{2}$ statistical test, we were not able to reject the null hypothesis ($\chi^{2}=600.46,\;df=4,\;p<0.05$) that there was a dependency between the therapist’s and ASPECT’s score.

### 4.2. Comparison between Therapist and ASPECT Scores by Age

When the age groups were considered, the results were consistent with the overall findings, especially for ages 4 and 6. There was only one 5-year-old participant who had a medium starting EESA score. In that case, the therapist and ASPECT agreed on 43 (57%) out of 75 observations due to a disagreement on utterances that were scored “correct” by ASPECT and “incorrect” by the therapist. There was also one 7-year-old participant with a high starting EESA score in the study. In that case, ASPECT and the therapist agreed on 81 (96%) out of 84 utterances. We failed to reject the null hypothesis for all age groups (p<0.05) that there was a dependency between the therapist’s and ASPECT’s scores.

The accuracy of ASPECT tended to increase with the age of the participant. The likely explanation for this trend is the positive correlation between the echoic proficiency and age in our participants.

### 4.3. Comparison between Therapist and ASPECT Scores by Pre-Treatment EESA Score

When the participants were divided into three groups based on their pre-treatment EESA score, more distinctive differences were observed. Although there was an overall agreement in 130 (75%) out of 173 observations, ASPECT identified fewer utterances as “correct”, 1 (1%) out of 173, when compared to the therapist with participants whose pre-treatment EESA was lower than 50 (Table 2). Similarly, in the group whose EESA score was between 50 and 75, ASPECT classified 8 (19%) out of 43 “correct” responses as “incorrect” or “no response” (Table 3).

ASPECT and the therapist agreed on 403 (69%) out of 703 scores in the case of participants with a pre-treatment EESA score of 75 and above (Table 4). Participants belonging to the group with high EESA contributed 703 (72%) out of 979 observations. We also failed to reject the null hypothesis (*p* < 0.05) that there was a dependency between the therapist’s and ASPECT’s scores for all EESA score levels.

## 5. Discussion

Among nine children diagnosed with ASD and enrolled in the local autism support center, we showed that a custom-built digital voice assistant-based application named ASPECT provided a classification of their utterances that exhibit statistical dependency when compared to the therapist’s scores. We assume that the therapist was always correct in their scoring and, thus, used their score as the baseline for ASPECT’s accuracy.

ASPECT performed well for participants with low echoic skills (EESA lower than 50) and those with a high EESA score (EESA greater and equal to 75) in matching “correct,” “incorrect,” and “no response” scores from the therapist. The assumption is that the group with a low starting EESA score had a lot of “incorrect” and “no responses,” which drove the high degree of agreement between ASPECT and the therapist. ASPECT performed less well for those in the middle tier of echoic skills (EESA between 50 and 75).

We attribute this drop in performance to the fact that ASPECT follows an intent-based model. As a result, it tended to have difficulty when (1) the utterance was not loud enough to be picked up by its sensors but the therapist could hear it, (2) the utterance had no relevance to the session but was picked up as an intent by ASPECT. The therapist scored that response as “no response”, while ASPECT marked it “incorrect”.

The accuracy of ASPECT for participants with high echoic skills was like the overall performance of ASPECT. We can attribute this to the fact that participants in this group already have good verbal and echoic skills, and the intent model could determine the outcome more easily.

In some observations, ASPECT scored an utterance as “correct” when the therapist deemed it as “no response” or “incorrect.” In those cases, ASPECT could pick up that there was an utterance and could match it to the expected intent even if the therapist disagreed on the scoring. Over time, we could see the emergence of a common language that only participants with a particular echoic skill and ASPECT-like applications that were trained to that participant could understand. This is like the emergence of a dialect where continuous use of an ASPECT trains its model to learn the language of the participant.

This phenomenon can be both positive and negative. For participants whose echoic skills cannot be improved, a digital voice assistant could provide support in independent living by allowing them to communicate their needs for shopping, movies, and online activities. ASPECT can also act as a translator between the participant and people who have difficulties understanding their utterances. On the negative side, consistent use of ASPECT can stunt the development of echoic skills in participants by allowing them to communicate more easily with the ASPECT, thus, impeding the natural shaping process of language development.

In our study, the subjects were a subgroup of the population, i.e., children with cognitive and sensory challenges. In that context, we need to acknowledge that ASR’s performance heavily depends on the training data [35]. For example, racial disparities were found in ASRs developed by Amazon, Apple, IBM, Google, and Microsoft [36]. Similarly, speech pathologies and impairment are also challenges for acoustic models [35]. Therefore, the inherent biases can impact ASPECT performance compared to the therapist’s evaluation of the utterances spoken by the children with ASD. This leads to the conclusion that more systematic studies aimed at quantifying the bias of ASRs against people on the spectrum are needed.

### Limitations

Since this was a cohort study, we cannot demonstrate causality, as confounding variables could be influencing the results. A randomized controlled trial (RCT) would have been the best study design to test our approach. However, caregiver- or therapist-administered RCTs focusing on computer-supported therapy are reported to have a high attrition rate [19,37]. We also had access to a single therapy center with a significant rotation in the center’s staff, leading to a high attrition rate among therapists involved in the data collection process. We also needed to provide the center with ten Echo Dot Devices, and since our study was self-funded, we could not scale this to more locations.

This study also has limitations inherent to the technology used in data collection. The Alexa Echo Dot device has a built-in array of microphones to increase its sensitivity to spoken commands. However in many instances, participants spoke too quietly for the device to pick up their utterances. Other sounds, like a moving chair and objects moved by the child, can also interfere with the device’s ability to pick up utterances. Second, there were nine participants, and most of them belonged to the group characterized by high echoic skills (703 (72%) out of 979 observations).

We can see that the distribution of scores varied across the groups characterized by EESA, and this could change if more responses were collected from participants with low and medium echoic skills. Lastly, Amazon and its digital voice assistants linked to the AWS framework are only one of several vendors. It is possible that other devices and software technology would perform differently.

## 6. Conclusions

IoT technology has the potential to assist people with disabilities by removing artificial barriers preventing them from living independent and productive lives. Omnipresent digital voice assistants, such as Amazon’s Alexa, can provide therapeutic opportunities to people with disorders, such as ASD, impeding verbal behavior (e.g., echoics, mands, and tacts). This paper reported a study focusing on determining how accurately the Amazon Alexa-based skill we developed, called ASPECT, recognized utterances spoken by an autistic child. The study conducted at the local therapy center involved nine children diagnosed with ASD.

We established that ASPECT scored the participants in the same way the therapist did in almost 7 out of 10 cases, thus, showing a statistical dependency. In addition, ASPECT performed exceptionally well when the assessed echoic skills were low or high. Children with more lacking echoic skills produced more incorrect and no responses, which appeared to be easier for ASPECT to classify in the same way the therapist did. Children with higher echoic skills provided more correct responses, which contributed to the higher accuracy of ASPECT.

The results suggest that some children, while understood by ASPECT’s intent model, did not meet the criteria for the utterance to be accepted as a “correct” by the therapist. This indicates that DVA-based and, more broadly, IoT-based solutions could be used to interface systems enabling independent living. It also suggests that a therapist’s scoring criteria could be stricter than those needed to lead an independent and productive life.

Another conclusion stems from the fact that some responses scored by the therapist as “correct” or “incorect” were scored by ASPECT as “no response”. This suggests that if the device’s microphone array was more sensitive or equipped with more transducers, it could pick up the correctly spoken phrase, thereby, increasing the overall accuracy and utility.

In summary, ASPECT’s classification accuracy of the utterances spoken by children diagnosed with ASD during vocalizations and echoic responding sessions showed significant potential for providing a continuum of therapeutic opportunities outside the clinic. These devices could also enable a better understanding of how much time a student spends on developing language skills. More broadly, the ability to develop language skills can enable people on the extremes of the sensory and cognitive spectra to live independently by allowing them to communicate their needs and wants.

## Figures and Tables

**Table 1:** ASPECT vs. therapist scoring of utterances across 30 sessions (*N* = 979).

	ASPECT	Correct	Incorrect	No Response
Therapist				
Correct		203 (21%)	54 (5%)	50 (5%)
Incorrect		65 (7%)	177 (18%)	103 (10%)
No Response		7 (1%)	25 (3%)	295 (30%)

**Table 2:** ASPECT vs. therapist scoring of utterances for participants with low EESA (EESA < 50) across 30 sessions (N=173).

	ASPECT	Correct	Incorrect	No Response
Therapist				
Correct		1 (1%)	12 (7%)	1 (1%)
Incorrect		14 (8%)	87 (50%)	8 (5%)
No Response		0 (0%)	8 (5%)	42 (24%)

**Table 3:** ASPECT vs. therapist scoring of utterances for participants with medium EESA (50 ≤ EESA < 75) across 30 sessions (*N* = 103).

	ASPECT	Correct	Incorrect	No Response
Therapist				
Correct		35 (34%)	12 (7%)	10 (10%)
Incorrect		3 (3%)	8 (8%)	12 (12%)
No Response		5 (5%)	1 (1%)	17 (16%)

**Table 4:** ASPECT vs. therapist scoring of utterances for participants with high EESA (EESA ≥ 75) across 30 sessions (*N* = 703).

	ASPECT	Correct	Incorrect	No Response
Therapist				
Correct		167 (24%)	30 (4%)	39 (5%)
Incorrect		48 (7%)	82 (12%)	83 (12%)
No Response		2 (0%)	16 (2%)	236 (34%)

## Data Availability

The data obtained during the study is available at https://osf.io/3w5yf/ (accessed on 1 June 2021).

## Supplementary Materials

The following are available at https://www.mdpi.com/article/10.3390/s21134621/s1, Table S1: Application of guidelines for developing interactive software for children with ASD to ASPECT based on [26]; The list of utterances used to train ASPECT’s intent model.

## Abbreviations

The following abbreviations are used in this manuscript:

AI	Artificial intelligence
ASD	Autism spectrum disorder
ASR	Automatic speech recognition
ASPECT	Autism Spectrum Engaged Cloud Technology
DVA	Digital voice assistant
EESA	Early Echoic Skills Assessment
MITA	Mental Imagery Therapy for Autism
PSF	Prefrontal synthesis
RCT	Randomized controlled trial
SLP	Speech-language pathologist
VB-MAPP	The Verbal Behavior Milestones Assessment and Placement Program
