# Supplementary material for: Internet-of-Things Devices in Support of the Development of Echoic Skills among Children with Autism Spectrum Disorder
Source: Sensors (Basel). 2021 Jul 5;21(13):4621. doi: 10.3390/s21134621 (PMC8272129; doi:10.3390/s21134621)
Supplement: Supplementary file 1 [file sensors-21-04621-s001.zip › sensors-1259193-supplementary.pdf]

The list of utterances used to train ASPECT's intent model:

3-4 shut the door
a big apple
a big apple tree
a big mud puddle
a cow says moo-moo
a good book
a really big puddle
anyway
apple
apple tree
are you done
at the end
babe
baby
bagels
ball
banana
bare
barney
bat
bath
bathtub
bear
bed
bee
big table
bike
binky
binky boo
bologna
bologna sandwich
bone
bones and muscles
boo
book
bow
bow wow
bowl
boyfriend
breakfast and lunch
broken
brown
brown puppy
brush
go away
go bye bye
go bye-bye
goat
goldfish
goldfish crackers
goofy
goofy goat
grapes
halloween treat
hammer
hammer and nails
hankie
happy
hat
helicopter
high
hike
holiday fun
hot dog
how
how many
how much do you want
hug
I like bagels
I like computers
I like to play sports
I want
I want a break
I want a long break
I want a puppy
I want a small puppy
I want juice
I want pecan ice cream
I want some ice cream
I want to go
I want yogurt
ice
ice cream
icky
I'm all done
in the swimming pool
is los
itches
pine
pizza
plane
pokey
pool
pop
potato
potty
price
prize
pudding
pup
puppet
puppet game
puppy
put bubbles in the tub
puzzle
refrigerator
rice
running
sandwich
school
schoolbus
see
see tuba
shine
shirt
shoe
sit
skittle
slide
slider
soda
spaghetti
spoon
strawberries and cream
swimming
swimming pool
syrup
table
taco
teacher
ted
teddy

brush my hair
bubble
bubblegum
bubbles
bubbles in the tub
bumble
bumble bee
bun
bunny
buzz
buzzer
bye
bye-bye
cake
can I go
can I go outside now
can I go potty
can you fix it please
car
cat
cheese
cheese and crackers
chips
chocolate pudding
climbing
coloring
coloring book
come up
come up with a guess
cookie
cookie monster
cover me u
crackers
cried
cry
cup
daddy
dentist
dimes and quarters
dip
do you play
do you play soccer too
do you want some too
doctor
dog
it's in the swimming pool
juice
king
kit
kitty
kitty cat
knife
lakehouse
lego
let the water out
let's color
let's color the picture
let's go home
let's read
let's read a good book
little brown puppy
look at my new snow boots
lost
macaroni
make a bowl
make a bowl of oatmeal
many
medicine
meow
mice
might
milk
milk and cookies
mitten
mom
mom and dad
mommy
money lender
monk
monkey
monster
moo
movie
mud puddle
my fries
my little brown puppy
my new pencil is lost
my nose itches
my pencil is lost
my puppy
teddy bear
tell it like it is
the big piece of cake
the big sun
the big sun is yellow
the cat is climbing
the clown's
the clown's face
the clown's funny face
the clown's funny red face
the dog
the dog is barking
the dog is brown
the library's closed
the man is driving
the pencil
the water is running
this is my nose
three
three four shut the door
tickle
tide
time to go
time to go to bed
tired
tomato
tonight
too
toy
treat
tree
trick
trick or treat
truck
tub
tuba
tubby
tubby toy
tummy
turn left at the corner
TV
twelve
two puzzles
uh-oh
umbrella

doggy
doggy bone
doll
don’t
don’t let the water out
down
drink
drive
driving
eat
eight
fat
fat doggy
fire
flat
flip me upside down
fold the map
food
forget about it now
fork
fried
fun
funny
funny king
game
get my blue pajamas
get my pajamas
girlfriend
give me pizza
go
my teacher
my tummy hurts
nails
newspaper
nighttime
no
north is up
nose
numbers tell the tale
nut
oatmeal
octopus
oh
okay
one
open
open the book
open the book now
open up
ouch
outside now
pajamas
pancakes
pancakes and syrup
panda
pay
pea
peanut
peanut hat
pencil
under the big table
under the table
under the wood table
up
up in the air
video
video tape
water
water is running
wet
wet mitten
where did the man go
where is big bird
where is my teacher
whine
window
winnie the pooh
wipe
wonder
wonderful
wow
yellow
yes
yogurt
you won the game
yuck
yucky
yumm-o
yummy
yummy food

Table S1: Application of guidelines for developing interactive software for children with ASD to ASPECT.

Guideline	Description
1	The team members without the therapeutic experience visited the therapy center to observe children during their activities. In addition, video recordings of the sessions aimed at practicing echoic skills were watched.
2	ASPECT replicated practice sessions aimed at improving echoic skills. The therapist selected utterances based on the child's abilities and the intended objectives of the session.
3	The study and the recruitment script were approved by the IRB. Each guardian was provided a consent form, while children were provided an assent form (if applicable).
4	ASPECT replicated practice sessions aimed at improving echoic skills. We allowed for practice sessions familiarizing the child with ASPECT.
5	All children whose data were reported completed 30 sessions. Data from children who had not completed 30 sessions or expressed maladaptive behavior were excluded from the study.
6	A session with ASPECT ended in the same way a regular session with a therapist did.
7	To maintain ecological validity, no special arrangements were made.
8	The sessions were conducted by the therapist who was part of the research team.
9	For child's comfort, only their therapist was present during the sessions with ASPECT.
10	Sessions with ASPECT were limited to 5 minutes. The therapist was allowed to end a session with ASPECT at any point (e.g., child's discomfort, maladaptive behavior).
11	The sessions with ASPECT were part of the overall therapy plan. It inherently had the same flexibility as the regular sessions.
12	To maintain ecological validity, no changes to the room were made. The only new object was the Echo Dot used in the study. Many of the children were familiar with the device, commonly seen in their homes and the center.
13	ASPECT replicated practice sessions aimed at improving echoic skills. It mimicked the way a therapist would interact with the child during a session.
14	Our study was conducted in the same spaces where the child had their regular sessions with the therapist.
15	ASPECT replicated practice sessions aimed at improving echoic skills. It mimicked the way a therapist would interact with the child during a session. No additional stimuli were present.
16	This version of ASPECT did not offer customization or adaptation other than utterances matching the child's abilities and therapeutic objectives set by the therapist.
17	No special interests and fears of the child were accommodated in this version of ASPECT.
18	ASPECT allowed only the limited number of attempts per utterance. Children had no control over ASPECT.
19	ASPECT was logging data to the extent permitted by the IRB.
20	Even if a child failed to speak an utterance, ASPECT provided encouraging feedback.
